# Staggered-peak production is a mixed blessing in the control of particulate matter pollution

**DOI:** 10.1038/s41612-022-00322-x

**Published:** 2022-12-10

**Authors:** Ying Wang, Ru-Jin Huang, Wei Xu, Haobin Zhong, Jing Duan, Chunshui Lin, Yifang Gu, Ting Wang, Yongjie Li, Jurgita Ovadnevaite, Darius Ceburnis, Colin O’Dowd

**Affiliations:** 1 State Key Laboratory of Loess and Quaternary Geology, Center for Excellence in Quaternary Science and Global Change, Institute of Earth Environment, Chinese Academy of Sciences, Xi’an, 710061 China; 2 Interdisciplinary Research Center of Earth Science Frontier (IRCESF), Beijing Normal University, Beijing, 100875 China; 3 Laoshan Laboratory, Qingdao, 266061 China; 4 University of Chinese Academy of Sciences, Beijing, 100049 China; 5 Ryan Institute’s Centre for Climate & Air Pollution Studies, School of Natural Sciences, Physics Unit, University of Galway, University Road, Galway, H91CF50 Ireland; 6 Department of Civil and Environmental Engineering, Faculty of Science and Technology, University of Macau, Taipa, Macau, SAR 999078 China

**Keywords:** Environmental impact, Atmospheric chemistry

## Abstract

Staggered-peak production (SP)—a measure to halt industrial production in the heating season—has been implemented in North China Plain to alleviate air pollution. We compared the variations of PM_1_ composition in Beijing during the SP period in the 2016 heating season (SP_hs_) with those in the normal production (NP) periods during the 2015 heating season (NP_hs_) and 2016 non-heating season (NP_nhs_) to investigate the effectiveness of SP. The PM_1_ mass concentration decreased from 70.0 ± 54.4 μg m^−3^ in NP_hs_ to 53.0 ± 56.4 μg m^−3^ in SP_hs_, with prominent reductions in primary emissions. However, the fraction of nitrate during SP_hs_ (20.2%) was roughly twice that during NP_hs_ (12.7%) despite a large decrease of NO_x_, suggesting an efficient transformation of NO_x_ to nitrate during the SP period. This is consistent with the increase of oxygenated organic aerosol (OOA), which almost doubled from NP_hs_ (22.5%) to SP_hs_ (43.0%) in the total organic aerosol (OA) fraction, highlighting efficient secondary formation during SP. The PM_1_ loading was similar between SP_hs_ (53.0 ± 56.4 μg m^−3^) and NP_nhs_ (50.7 ± 49.4 μg m^−3^), indicating a smaller difference in PM pollution between heating and non-heating seasons after the implementation of the SP measure. In addition, a machine learning technique was used to decouple the impact of meteorology on air pollutants. The deweathered results were comparable with the observed results, indicating that meteorological conditions did not have a large impact on the comparison results. Our study indicates that the SP policy is effective in reducing primary emissions but promotes the formation of secondary species.

## Introduction

Particulate matter (PM) pollution exerts profound impacts on human health^1–5^, climate^6–8^, visibility^9,10^, and ecosystem^11^. Due to rapid industrialization and urbanization over the last few decades, China has suffered from persistent and pervasive haze pollution, especially during winter^12–15^. As one of the largest megacities in China, Beijing has undergone serious air pollution over the past decades^13,15,16^. The annual PM_2.5_ loadings ranged from 89.5 to 73.0 μg m^−3^ during 2013–2016 in Beijing (Beijing Municipal Ecology and environment Bureau, http://sthjj.beijing.gov.cn/bjhrb/), exceeding the Chinese National Ambient Air Quality Standard (CNAAQS, 35 µg m^−3^). In recent years, various stringent pollution prevention and control measures covering main pollution sectors have been implemented nationwide to alleviate air pollution, for example, traffic restriction^17^, the coal-to-gas shift^18^, and the Air Pollution Prevention Action Plan^19^. There are many studies investigating the effectiveness of these air quality interventions. For example, Gao et al. found a 21% decrease in average PM_2.5_ concentration in Beijing during winters from 2011 to 2016, which was mainly attributed to stringent emission control measures^20^. Gu et al. illustrated that the average concentrations of PM_1_ in urban Beijing in 2014–2015 decreased by 16-43% compared to those in 2008–2013 after the implementation of emission control measures since 2013^21^. The size distribution, oxidation properties, and acidity of aerosols changed substantially after the implementation of the clean action plan^22–24^. In addition, short-term emission controls are also effective in improving air quality. Large reductions of concentrations in the major components of submicron aerosol have been reported during the 2014 Asia-Pacific Economic Cooperation (APEC) summit^25^. The mass concentration of PM (PM_1_) decreased by 57% due to stringent control during the China Victory Day parade in 2015^26^. These results demonstrate that air pollution has been effectively mitigated after stringent control. Specifically, meteorological conditions also can affect the variation of pollutant concentration, which makes it difficult to directly compare the pollutant emission levels. Zhang et al. estimated that meteorological conditions contributed to 9% of the national PM_2.5_ reduction from 2013 to 2017 and contributed to 16% of the Beijing-Tianjin-Hebei region (BTH) PM_2.5_ reduction from 2013 to 2017^27^. In contrast, during the COVID-19 lockdown period, severe haze pollution was facilitated by stagnant meteorology and high RH despite the substantial reduction of primary emissions^28–30^. Thus, it is essential to decouple the meteorological impacts from ambient air quality to evaluate the effectiveness of the control measures in Beijing. Regression models^31,32^, chemical transport models^20,33–35^, and machine learning models are common methods to decouple the potential effects of weather-related variations^36–40^. Detailed comparisons of these methods can be found elsewhere^37,39,41^. The machine learning-based random forest (RF) algorithm showed high prediction accuracy by reducing variance and error in high dimensional data sets, and the learning process can be explained and interpreted where the importance of input variables and their interactions are visualized^37,41^. Grange et al. applied a meteorological normalization technique based on the RF algorithm to control changes in meteorology when conducting air quality data analysis^36^. Shi et al. used a machine learning-based RF algorithm to evaluate major reductions in air pollutant emissions after the short-term emission interventions^39^.

Despite effective mitigation of air pollution, Beijing still suffers severe pollution in winter. The municipalities of Beijing issued a convention on halting cement production, which was fully implemented in North China during the heating season in 2015 to improve the air quality and to reduce the cement production overcapacity (https://wap.miit.gov.cn/). However, the effectiveness of this policy, named staggered-peak production (SP), in mitigating PM pollution is yet to be evaluated. Therefore, it is of great significance to investigate the impact of SP measures on the atmospheric environment. In this study, an aerosol chemical speciation monitor (ACSM) and an aethalometer were deployed to measure the composition of PM_1_. The aerosol chemical composition of PM_1_ in Beijing in 2015 and 2016, which covers the periods before, during, and after implementing the SP policy, are investigated, and the observation results are further compared with the deweathered results by using machine learning techniques. Moreover, the causes of secondary formation and variations of PM_1_ composition under different pollution stages after the SP are explored with detailed analysis.

## Results

### General changes in PM_1_ pollution

**Fig. 1:**
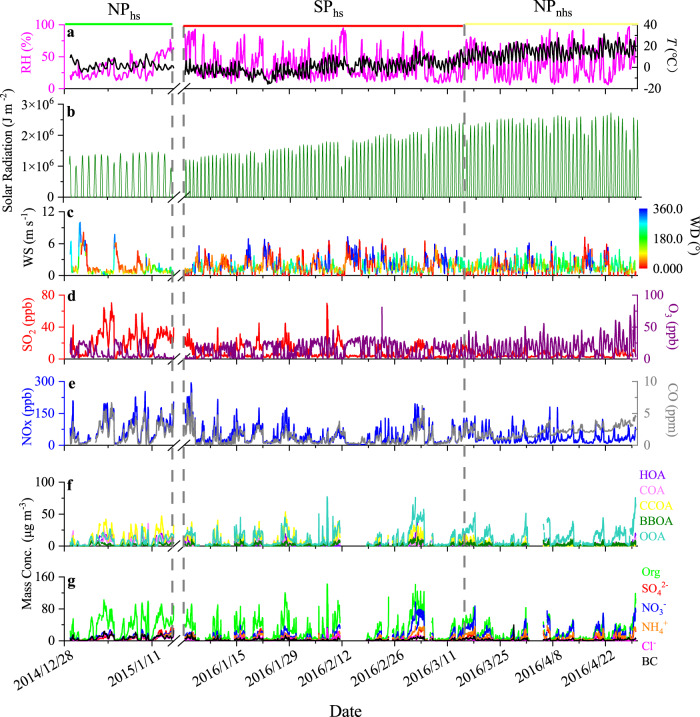
Time series of PM_1_ species, OA factors, gaseous precursors, and meteorological parameters for the NP_hs_, SP_hs_ and NP_nhs_. Time series of **a** temperature (*T*) and relative humidity (RH), **b** surface net solar radiation, **c** wind speed (WS) and wind direction (WD), **d** SO_2_ and O_3_, **e** NOx and CO, **f** OA factors (HOA, COA, CCOA, BBOA, and OOA), **g** PM_1_ species (organic, sulfate, nitrate, ammonium, chloride and black carbon) for the NP_hs_, SP_hs_ and NP_nhs_. NP_hs_ represent the normal production period in the heating season from December 29th, 2014, to January 14th, 2015, SP_hs_ represents the staggering-peak production period in the heating season from January 1st, 2016, to March 15th, 2016, and NP_nhs_ represents the normal production period in the non-heating season from March 16th, 2016 to April 30th, 2016.

The SP measure on the cement industry was implemented in Beijing from January 15th, 2015, to March 15th, 2015, as well as from November 15th, 2015, to March 15th, 2016, which overlapped for several months with our measurement conducted from December 29th, 2014 to January 14th, 2015 and from January 1st, 2016 to April 30th, 2016. To investigate the effects of the SP measure on PM_1_ characteristics, the entire campaign was split into three periods, including the SP period (staggered-peak production period) in the heating season, the NP period (normal production period) in the heating season, and the NP period in the non-heating season. Five OA factors, including hydrocarbon-like OA (HOA), cooking OA (COA), coal combustion OA (CCOA), biomass burning OA (BBOA), and oxygenated OA (OOA), were resolved in NP_hs_ and six OA factors including HOA, COA, CCOA, BBOA, local secondary OA (LSOA) and regional secondary OA (RSOA) were resolved in SP_hs_ and NP_nhs_. OOA during SP_hs_ and NP_nhs_ is the sum of LSOA and RSOA for a better comparison with NP_hs_. Details of OA source apportionment are shown in the Method section. Periodic pollution events occur sporadically, with the mass concentration of PM_1_ ranging from 3.0 to 201.8 μg m^−^^3^ in NP_hs_, 2.4 to 305.7 μg m^−3^ in SP_hs_, and 2.9 to 274.1 μg m^−3^ in NP_nhs_, respectively (as shown in Fig. 1). The impact of meteorology was normalized by using a machine learning technique because the variations of PM_1_ species and OA factors are affected by meteorological conditions, for example, heavy pollution episodes were related to southerly winds with low wind speeds (<2 m s^−1^) and high relative humidity (RH).

### Observed and deweathered changes in primary emissions and secondary formation

**Fig. 2:**
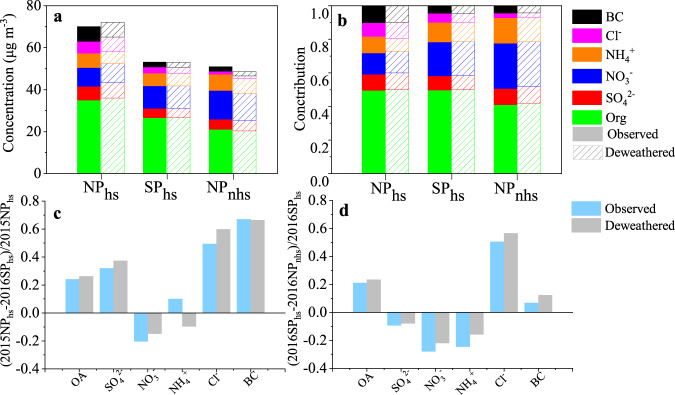
Comparisons of PM_1_ species between deweathered and observed results. **a** Mass concentrations and **b** fractions of deweathered and observed PM_1_ species during the NP_hs_, SP_hs_, and NP_nhs_. The observed and deweathered change ratios of PM_1_ species between **c** NP_hs_ and SP_hs_, **d** SP_hs_ and NP_nhs_ (the bars below the horizontal line represent increased ratios, and the bars above the horizontal line represent decreased ratios of PM species).

The average mass concentration of PM_1_ in SP_hs_ (53.0 ± 56.4 μg m^−^^3^) was much lower than that in NP_hs_ (70.0 ± 54.4 μg m^−^^3^) (Fig. 2 and Table 1), pointing that SP is potentially effective in reducing PM. The primary emissions of PM_1_ components and OA factors, including chloride, black carbon (BC), HOA, COA, and CCOA, decreased by 36.1–66.9% from NP_hs_ to SP_hs_. In particular, CCOA decreased by 4.5 μg m^−3^, which was the most among OA factors. Given that coal is an important energy source for both industrial and residential heating, part of the reduction of CCOA was likely due to the SP measure. As for secondary species, sulfate (from 6.6 ± 7.3 to 4.5 ± 5.7 μg m^−3^) and ammonium (from 6.9 ± 5.8 to 6.2 ± 7.0 μg m^−3^) also decreased slightly from NP_hs_ to SP_hs_, with decreasing SO_2_ from 21.6 ± 14.9 ppb in NP_hs_ to 9.2 ± 8.2 ppb in SP_hs_. However, it should be noted that despite a large decrease of NO_*x*_ from 77.3 ± 54.6 ppb to 44.0 ± 42.6 ppb, nitrate mass concentration increased from 8.9 ± 7.4 μg m^−3^ in NP_hs_ to 10.7 ± 13.9 μg m^−^^3^ in SP_hs_, with its mass fraction in PM_1_ consequently increasing from 12.7% in NP_hs_ to 20.2% in SP_hs_. This phenomenon may be due to stronger atmospheric oxidation capacity and higher nitrogen oxidation ratio in SP_hs_, and will be further discussed in the following section. OOA was the most abundant OA factor in SP_hs_, on average accounting for 43.0% of OA, which was much higher than that during NP_hs_ (22.5%). These variations suggest reduced primary emissions and enhanced secondary aerosol contributions in SP_hs_ after the implementation of the SP measure. Similar findings were observed by Huang et al.^30^ and Wang et al.^42^, but variations of oxygenated organic aerosol and influences of meteorological parameters were not considered in those two studies.

**Table 1:** Observed mass concentrations (averages + standard deviations) of aerosol species and OA factors, gaseous pollutants, and meteorological parameters during three different periods.

Species	NP_hs_	SP_hs_	NP_nhs_
*Aerosol species (μg* *m*^*−3*^*)*			
PM_1_	70.0 ± 54.4	53.0 ± 56.4	50.7 ± 49.4
OA	34.8 ± 26.8	26.4 ± 27.1	20.8 ± 18.2
HOA	6.1 ± 5.6	2.4 ± 4.0	1.9 ± 2.6
COA	6.5 ± 5.6	2.7 ± 2.7	1.9 ± 1.9
CCOA	12.5 ± 11.3	8.0 ± 9.0	1.9 ± 2.8
BBOA	1.9 ± 1.8	2.0 ± 2.5	1.9 ± 1.9
OOA	7.8 ± 6.7	11.3 ± 12.5 (LSOA:10.0 ± 11.4; RSOA:1.3 ± 1.7)	13.2 ± 11.9 (LSOA:10.2 ± 10.3; RSOA: 3.0 ± 2.2)
SO_4_^2−^	6.6 ± 7.3	4.5 ± 5.7	4.9 ± 6.5
NO_3_^−^	8.9 ± 7.4	10.7 ± 13.9	13.7 ± 16.6
NH_4_^+^	6.9 ± 5.8	6.2 ± 7.0	7.7 ± 7.8
Cl^−^	5.7 ± 5.1	2.9 ± 3.7	1.4 ± 1.7
BC	7.1 ± 5.6	2.3 ± 2.6	2.2 ± 2.3
*Gaseous pollutants*			
SO_2_ (ppb)	21.6 ± 14.9	9.2 ± 8.2	5.2 ± 3.1
CO (ppm)	1.9 ± 1.5	1.1 ± 1.1	2.2 ± 0.8
NO (ppb)	37.4 ± 37.5	17.7 ± 28.2	10.0 ± 17.4
NO_2_ (ppb)	39.9 ± 21.3	26.3 ± 18.0	27.4 ± 14.4
O_3_ (ppb)	9.8 ± 9.0	14.1 ± 11.2	17.9 ± 13.7
*Meteorological parameters*			
RH (%)	31.7 ± 14.9	35.2 ± 19.8	38.1 ± 21.6
*T* (°C)	1.8 ± 3.5	-0.2 ± 6.0	14.8 ± 5.6
WS (m s^−1^)	1.7 ± 1.8	1.9 ± 1.3	1.8 ± 1.1

Comparing observation results between SP_hs_ and NP_nhs_, the average mass concentration of PM_1_ in SP_hs_ (53.0 ± 56.4 μg m^−3^) was similar to that (50.7 ± 49.4 μg m^−3^) in NP_nhs_. This is different from previous studies in that PM_1_ in the heating season was much higher than that in the non-heating season^43,44^. While it is interesting to note that the PM_1_ concentrations were comparable between SP_hs_ and NP_nhs_, gaseous parameters and aerosol composition varied considerably. For example, SO_2_ decreased by 43.5% from 9.2 ± 8.2 ppb in SP_hs_ to 5.2 ± 3.1 ppb in NP_nhs_. The concentrations of NO_2_ were comparable (26.3 ± 18.0 ppb versus 27.4 ± 14.4 ppb) during the two periods. Specifically, CO concentration nearly doubled (from 1.1 ± 1.1 ppm to 2.2 ± 0.8 ppm) from SP_hs_ to NP_nhs_, mainly due to the resumption of industrial production. O_3_ increased by 27.0% (from 14.1 ± 11.2 ppb in SP_hs_ to 17.9 ± 13.7 ppb in NP_nhs)_ with the increase of solar radiation and temperature (from 436776.5 ± 638447.0 J m^−^^2^ to 727050.6 ± 907261.9 J m^−^^2^ and from −0.2 ± 6.0 °C to 14.8 ± 5.6 °C)^45–47^. As for PM_1_ composition and OA factors, the primary species, including chloride, BC, HOA, COA, CCOA, and BBOA, decreased notably from SP_hs_ to NP_nhs_. Components related to coal combustion still showed dramatical decreases, e.g., the mass concentrations of chloride and CCOA decreased by 50.5% and 75.7% from SP_hs_ to NP_nhs_, respectively, indicating that there are still large emissions from coal combustion in SP_hs_, presumably from domestic heating that persist in SP_hs_. Comparatively, the relative contributions of secondary inorganic aerosol (SIA, nitrate, sulfate, and ammonium) in NP_nhs_ increased by 1.2–6.8% when compared to SP_hs_. Meanwhile, the mass fractions of LSOA and RSOA increased by 11.2% and 9.4% from SP_hs_ to NP_nhs_, respectively. These results indicated that the SP could effectively alleviate the PM pollution in the heating season, but the effects of seasonal variations and central heating were nonnegligible.

**Fig. 3:**
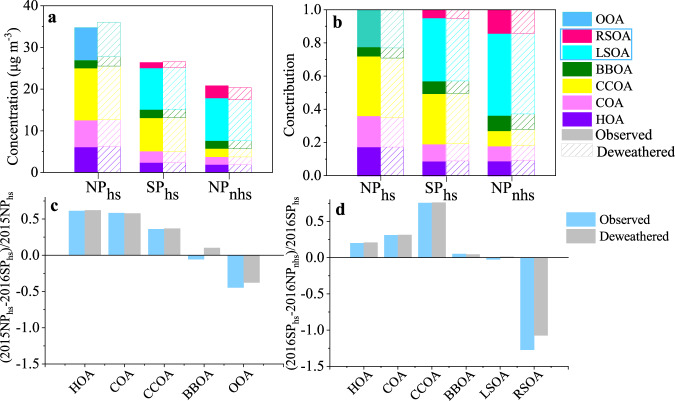
Comparisons of OA factors between deweathered and observed results. **a** Mass concentrations and **b** fractions of deweathered and observed OA factors during the NP_hs_, SP_hs_ and NP_nhs_. The observed and deweathered change ratios of OA factors between **c** NP_hs_ and SP_hs_, **d** SP_hs_ and NP_nhs_ (The bars below the horizontal line represent increased ratios and the bars above the horizontal line represent decreased ratios of OA factors).

To minimize the impact of the meteorological conditions on the above analysis, we apply the machine learning technique based on a random forest algorithm^37,39,41^ to obtain the deweathered concentrations of PM_1_ species, OA factors, and gaseous parameters (see Method for details). Smaller fluctuations were observed in the time series of the deweathered PM_1_ species and OA factors compared with observation results during the whole study (as shown in Supplementary Fig. 1). The average deweathered mass concentrations and fractional contributions of PM_1_ species and OA factors were comparable with the observations result in all three periods, as shown in Figs. 2, 3 and Table 2. The pollution load increased slightly (2.1 μg m^−^^3^, 0.1 μg m^−^^3^) after decoupling the effects of meteorology in NP_hs_ and SP_hs_, respectively. While in NP_nhs_, primary emissions and secondary formation reduced slightly after the weather normalization. After the weather normalization, the mass concentrations of PM_1_ still reduced largely from NP_hs_ to SP_hs_, and it still showed the characteristics of reduced primary emissions and enhanced secondary formation. The deweathered variations of PM_1_ species and OA factors from SP_hs_ to NP_nhs_ were similar to those observed. Even so, the reduction/increase ratios after decoupling the meteorological effects from NP_hs_ to SP_hs_ and from SP_hs_ and NP_nhs_ were somewhat different from those observed. For example, reductions of the deweathered OA, sulfate, and chloride from NP_hs_ to SP_hs_ (26.2%, 37.3%, 59.8%) were slightly larger than the reductions of observations (24.1%, 32.0%, 49.4%). The increases of deweathered nitrate and OOA from NP_hs_ to SP_hs_ were slightly lower than those observed. From SP_hs_ to NP_nhs_, the reductions of deweathered OA, chloride, and BC from SP_hs_ to NP_nhs_ were more pronounced than those observed, while the increase of SIA and OOA were lower than the observed results. In summary, the observed and deweathered results indicate reduced primary emissions but increased secondary formation during SP. Detailed causes of the enhanced secondary formation will be discussed in the following section.

**Table 2:** Deweathered mass concentrations (averages + standard deviations) of aerosol species and OA factors, gaseous pollutants, and meteorological parameters during three different periods.

Species	NP_hs_	SP_hs_	NP_nhs_
*Aerosol species (μg* *m*^*−3*^*)*			
PM_1_	72.1 ± 17.4	53.1 ± 13.5	48.7 ± 11.4
OA	36.0 ± 9.5	26.6 ± 6.9	20.4 ± 4.8
HOA	6.2 ± 2.0	2.4 ± 1.3	1.9 ± 0.7
COA	6.5 ± 2.3	2.7 ± 1.2	1.9 ± 0.8
CCOA	12.8 ± 4.3	8.1 ± 2.7	1.9 ± 1.7
BBOA	2.2 ± 0.7	2.0 ± 0.6	1.9 ± 0.3
OOA	8.3 ± 2.8	11.4 ± 3.0 (LSOA:10.0 ± 2.4; RSOA:1.4 ± 0.8)	12.8 ± 3.1 (LSOA:9.9 ± 2.9; RSOA: 2.9 ± 0.4)
SO_4_^2−^	7.2 ± 1.7	4.5 ± 1.0	4.9 ± 2.2
NO_3_^−^	9.3 ± 2.0	10.7 ± 4.1	13.0 ± 3.6
NH_4_^+^	6.9 ± 1.5	6.1 ± 1.5	7.1 ± 1.6
Cl^−^	5.6 ± 1.6	2.8 ± 0.8	1.2 ± 0.6
BC	7.1 ± 1.8	2.4 ± 1.0	2.1 ± 0.5
*Gaseous pollutants*			
SO_2_ (ppb)	21.1 ± 4.8	9.1 ± 1.9	5.3 ± 0.9
CO (ppm)	2.0 ± 0.5	1.1 ± 0.4	2.1 ± 0.6
NO (ppb)	39.2 ± 11.3	17.9 ± 12.7	10.9 ± 8.1
NO_2_ (ppb)	39.2 ± 6.6	26.4 ± 6.2	26.8 ± 4.4
O_3_ (ppb)	10.4 ± 3.9	14.2 ± 6.0	17.9 ± 9.6
*Meteorological parameters*			
RH (%)	31.7 ± 14.9	35.2 ± 19.8	38.1 ± 21.6
*T* (°C)	1.8 ± 3.5	−0.2 ± 6.0	14.8 ± 5.6
WS (m s^−1^)	1.7 ± 1.8	1.9 ± 1.3	1.8 ± 1.1

### The cause of enhancement of secondary formation

**Fig. 4:**
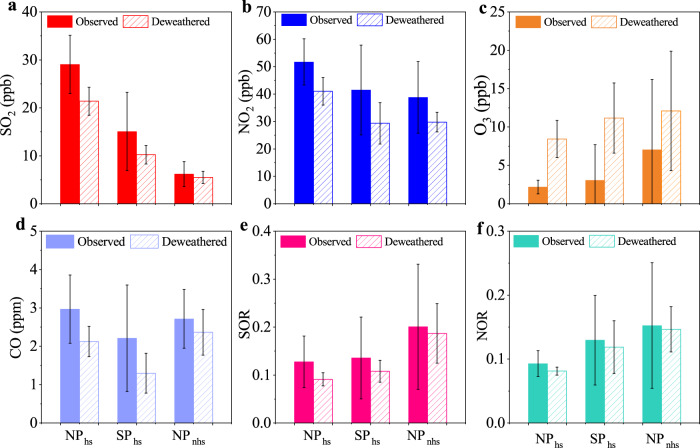
Observed and deweathered variations under high RH conditions (RH > 50%). Comparisons of observed and deweathered **a** SO_2_, **b** NO_2_, **c** O_3_, **d** CO, **e** SOR (sulfur oxidation ratio) and **f** NOR (nitrogen oxidation ratio) among NP_hs_, SP_hs_, and NP_nhs_. (Error bars represent the standard deviations of each species).

To further investigate secondary formation in different periods, the sulfur oxidation ratio (SOR = *n*[SO_4_^2−^]/(*n*[SO_4_^2^^−^] + *n*[SO_2_]) and nitrogen oxidation ratio (NOR = *n*[NO_3_^−^]/(*n*[NO_3_^−^] + *n*[NO_2_])^42,48,49^ were calculated. Considering that secondary formation was more pronounced under high RH conditions, data with RH > 50% was chosen for better comparison^16,50,51^. As shown in Fig. 4, the concentrations of precursors in SP_hs_ were 15.1 ± 8.2 ppb for SO_2_ and 41.5 ± 16.4 ppb for NO_2_, much lower than those in NP_hs_ (29.1 ± 6.1 ppb for SO_2_ and 51.7 ± 8.4 ppb for NO_2_). On the contrary, the concentration of O_3_ in SP_hs_ (3.1 ± 4.6 ppb) was higher than that in NP_hs_ (2.2 ± 0.9 ppb), indicating stronger atmospheric oxidation capacity in SP_hs_. The mass concentration of nitrate in SP_hs_ (20.9 ± 16.4 μg m^−3^) was 1.4 times that in NP_hs_ (15.1 ± 5.0 μg m^−3^), which was consistent with a higher NOR in SP_hs_ (0.13 ± 0.07) compared to that in NP_hs_ (0.09 ± 0.02). In comparison, the mass concentration of sulfate in SP_hs_ (10.7 ± 8.1 μg m^−3^) was lower than that in NP_hs_ (18.4 ± 8.0 μg m^−3^), which may relate to the lower SO_2_ concentration in SP_hs_ and similar SOR between SP_hs_ (0.14 ± 0.09) and NP_hs_ (0.13 ± 0.05). The concentrations of SO_2_ and NO_2_ decreased from 15.1 ± 8.2 ppb and 41.5 ± 16.4 ppb in SP_hs_ to 6.2 ± 2.6 ppb and 38.8 ± 13.1 ppb in NP_nhs_, respectively, while O_3_ largely increased from 3.1 ± 4.6 ppb in SP_hs_ to 7.0 ± 9.2 ppb in NP_nhs_ (as shown in Fig. 4). Consistently, the SOR and NOR in NP_nhs_ (SOR: 0.20 ± 0.13; NOR: 0.152 ± 0.10) were also higher than those in SP_hs_ (SOR: 0.14 ± 0.09; NOR: 0.13 ± 0.07). This was consistent with the increase of nitrate from SP_hs_ (20.9 ± 16.4 μg m^−3^) to NP_nhs_ (22.7 ± 19.2 μg m^−3^) (As shown in Supplementary Table 1). Specifically, the mass concentration of sulfate decreased from SP_hs_ (10.7 ± 8.1 μg m^−3^) to NP_nhs_ (8.1 ± 7.5 μg m^−^^3^), probably due to the reduction of SO_2_ from central heating emissions. These results suggested that the SP, central heating and seasonal variations all contributed to changes in secondary species.

As shown in Fig. 4 and Supplementary Table 1, SOR and NOR showed obvious decreases after decoupling the influence of meteorology, consistent with the prominent reductions of secondary species from observations to the weather normalization results. We noticed that observed SOR and NOR fell into wider ranges than the deweathered during the whole study period, indicating that the observed secondary formation was affected by various factors. Even so, deweathered SOR and NOR increased from NP_hs_ to SP_hs_ and increased from SP_hs_ to NP_nhs_, which were similar to the variational trends of observations, reaffirming secondary processes were stronger during the staggering peak production period in the heating season. The deweathered CO increased largely from SP_hs_ to NP_nhs_, indicating an increase in emissions from industrial production.

### Variations of PM_1_ composition under different pollution stages after the SP

**Fig. 5:**
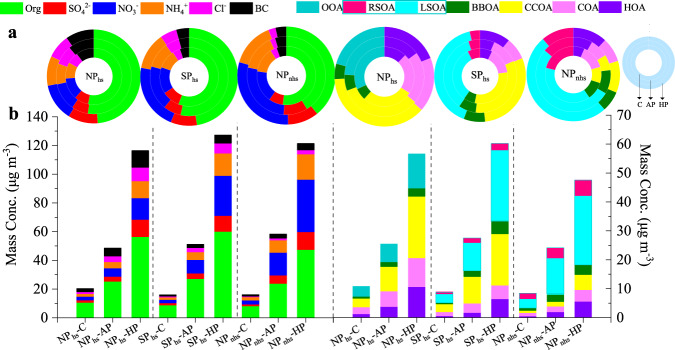
Variations of PM_1_ species and OA factors under different pollution stages. **a** Relative contributions and **b** average mass concentrations of PM_1_ species and OA factors on clean days (C), average-pollution days (AP), and heavy-pollution days (HP) during the NP_hs_, SP_hs_, and NP_nhs_ (As shown in the doughnut plot in the upper right corner, the innermost circle of this plot represents the clean days, the middle circle represents the average-pollution days, and the outermost circle represents the heavy-pollution days).

To further investigate the variations under different pollution stages after the implementation of SP, we divided the data into clean days (daily average PM_1_ < 35 µg m^−3^), average-pollution days (35 µg m^−3^ < daily average PM_1_ < 75 µg m^−3^), and heavy-pollution days (daily average PM_1_ > 75 µg m^−3^), respectively. As shown in Fig. 5, in NP_hs_, the relative contributions of chloride were the lowest on clean days (6.7%) when compared with the other two pollution stages (8.3% on average pollution days; 8.2% on heavy-pollution days). What’s more, the mass fractions of chloride in NP_hs_ were higher than those in SP_hs_ and NP_nhs_ in all pollution stages. The fractional contributions of POA to OA increased largely from 68.2% on clean days to 75.8% on average-pollution days and further to 79.2% on heavy-pollution days. As for secondary species, the fractional contribution of SIA in heavy-pollution days was the highest (33.4%) when compared with those on average-pollution days (28.0%) and clean days (29.8%), while the fractional contributions of OOA to OA decreased from 31.8% on clean days to 24.2% on average-pollution days and further to 20.8% on heavy-pollution days. These results indicated that primary emissions and secondary inorganic formations (e.g., nitrate and ammonium) contributed largely to heavy pollution events in NP_hs_. In SP_hs_, relative contributions of primary emissions were comparable in different pollution stages, while fractional contributions of secondary inorganic species increased from clean days (34.7%) to average-pollution days (36.4%) and further to heavy-pollution days (42.8%) and the increase ratios of SIA from clean days to average-pollution days and further to heavy-pollution days in SP_hs_ were larger than those in NP_hs_. LSOA presented a similar increasing trend with secondary inorganic species from clean days to heavy-pollution days. Specifically, the relative contributions of RSOA to OA decreased largely from clean days (7.4%) to average-pollution days (5.2%) and further to heavy-pollution days (3.7%). These results suggested that both primary emissions and secondary formation (e.g., nitrate, ammonium, and LSOA) were important in the haze formation in SP_hs_ and the secondary formation in SP_hs_ was stronger than that in NP_hs_. Different from NP_hs_ and SP_hs_, the mass fractions of chloride decreased from clean days (3.6%) to heavy-pollution days (2.4%) in NP_nhs_. The relative contributions of POA in NP_nhs_ also decreased from 40.3% on clean days to 38.4% on heavy-pollution days. However, fractional contributions of SIA increased largely from 39.1% on clean days to 51.9% on average-pollution days and further to 54.8% on heavy-pollution days in NP_nhs_. The relative contributions of LSOA to OA were also increased from clean days (38.5%) to heavy-pollution days (50.3%) in NP_nhs_. Although RSOA presented a similar decreasing trend with that in SP_hs_, the relative contributions of RSOA increased prominently from SP_hs_ and NP_nhs_ in all pollution stages. These results illustrated that when compared with NP_hs_ and SP_hs_, secondary formation, including local oxidation and regional transportation, was more prominent in aggravating atmospheric pollution in NP_nhs_.

## Discussion

In this study, we compared the chemical characteristics of PM_1_ in SP_hs_ with those in NP_hs_ and NP_nhs_ to evaluate the effectiveness of staggered-peak production measures. PM_1_ mass concentration in SP_hs_ decreased by about 24.3% compared to NP_hs_ with reduced primary emissions and enhanced secondary formation, indicating that the SP measure led to a substantial reduction of PM pollution in the heating season. The PM_1_ loading was similar between SP_hs_ (53.0 ± 56.4 μg m^−^^3^) and NP_nhs_ (50.7 ± 49.4 μg m^−3^), indicating reduced seasonal variations in PM pollution between heating and non-heating seasons after the implementation of SP. Specifically, the RF algorithm was used to decouple the effects of meteorological conditions. After decoupling the effects of meteorology, smaller fluctuations were observed in the time series of PM_1_ species and OA factors. Although the increase/decrease ratios varied substantially of PM_1_ composition and OA factors after weather normalization, the variation trends of primary emissions and secondary formations were consistent with the observed results, indicating that SP indeed alleviates PM pollution. Studies on different pollution levels show that secondary transformation is more important in the formation of haze events after the SP. These results call for further control of PM precursors and more investigations on secondary formation mechanisms under different conditions in highly polluted regions in urban China.

## Methods

### Sampling site

The sampling site is located in the northwest region of Beijing between the 4th and 5th ring roads, surrounded by various research institutes and residential areas (40.00N, 116.38E). Measurements were conducted on the rooftop of a five-story building of China’s National Center for Nanoscience and Technology (NCNST), about 20 m above the ground level. The campaign was conducted from January 1st to April 30th in 2016. Data in 2015 was used for comparison cited by Huang et al.^52^.

### Instrumentation

The composition of non-refractory submicron aerosol (NR-PM_1_ including organics, sulfate, nitrate, ammonium, and chloride, was measured by a quadrupole aerosol chemical speciation monitor (Q-ACSM, Aerodyne Research Inc., Billerica, Massachusetts) with unit mass resolution (UMR) and a time resolution of 30 minutes. A detailed operating principle of this instrument can be found elsewhere^53^. In brief, particles passing a URG cyclone (Model: URG-2000-30ED) with a cutting size of 2.5 μm were drawn through a 3/8 in. stainless steel tube at a flow rate of ~3 L min^−1^ and then entered the vacuum chamber of the instrument through an aerodynamic lens. Through the lens, aerosol particles with diameters of 40 nm–1 μm focused into a beam of particles and later vaporized at 600 °C and ionized with electron impact ionization. The ionized fragments then entered the mass spectrometer for detection and analysis. O_3_ and NO_*x*_ were measured by standard gas analyzers (Thermo Scientific, Model 42i, and 48i, respectively). The concentrations of CO and SO_2_ were also obtained by gas analyzers (ECOTECH, Model EC9803B, and EC9850B, respectively). The gas monitors were sampled in a time resolution of 5 min. Meanwhile, an aethalometer (model AE-33) was deployed to obtain the concentration of BC with a time resolution of 1 min. Note that here BC is from PM_2.5_ but is used to represent BC in PM_1_ as BC mass is mostly confined to the 10–1000 nm diameter range^42,54,55^. The meteorological data, including temperature (*T*), RH, wind speed (WS), and wind direction (WD), were obtained by an automatic weather station (MAWS201, Vaisala, Vantaa, Finland) and a wind sensor (Vaisala Model QMW101-M2).

### ACSM data analysis

Concentrations of the NR-PM_1_ species were analyzed using the standard ACSM software version 1.5.2.0 (Aerodyne Research Inc., Billerica, Massachusetts, USA). Following Ng et al.^56^, calibrations were conducted to ensure that the instrument was in good condition during the whole observation period. Specifically, an atomizer (Model 9302, TSI Inc., Shoreview, MN, USA), a differential mobility analyzer (DMA, TSI model 3080), and a condensation particle counter (CPC, TSI model 3772) were used for the calibration of ionization efficiency (IE) and the relative ionization efficiencies (RIEs). RIEs of organics, nitrate, chloride, ammonium, and sulfate were 1.4, 1.1, 1,3, 6.4, and 1.2, respectively. A composition-dependent collection efficiency (CDCE) was applied following Middlebrook et al.^57^, which is presented as max (0.45, 0.0833 + 0.9167×*ANMF*), ANMF is the mass fraction of ammonium nitrate in NR-PM_1_.

### OA source apportionment

Source apportionment was performed on the OA data using PMF with a multilinear engine (ME-2)^58^. Details of source apportionment of OA were provided in the Supplementary Information (Supplementary Note 1 and as shown in Supplementary Figs. 2-4). Briefly, we examined solutions from 2 to 8 factors using the unconstrained PMF model. According to the analysis of mass spectra, diurnal cycles, time series of each factor, and comparisons with factors from previous studies, we first interpreted five factors, which were hydrocarbon-like OA (HOA), cooking OA (COA), coal combustion OA (CCOA), oxygenated OA1 (OOA1) and oxygenated OA2 (OOA2). However, in the free PMF solution, COA and HOA were mixed as the COA profile had the alkyl fragments signatures, which were characteristics of HOA. Meanwhile, we found obvious signals for m/z 60 (mainly C_2_H_4_O_2_^+^) and m/z 73 (mainly C_3_H_5_O_2_^+^), which were considered BBOA tracers in HOA and CCOA factors. Besides, the fraction of the ion peak at m/z 60 (*f*60) makes up approximately 0.5% of organic matter mass, slightly larger than the environmental background value of 0.3%^59^, indicating the contribution of BBOA.

To separate the factors from mixtures, ME-2 was used to provide a complete exploration of the rotational ambiguity by introducing *a* priori information. The final result was the average of 33 solutions based on minimization of m/z 60 in HOA, optimization of COA diurnal patterns, and the consistency of factors with the previous studies^15,16,60,61^. OOA1 and OOA2 were further interpreted as local secondary OA (LSOA) and regional secondary OA (RSOA), which were described in detail in the Supplementary Information (As shown in Supplementary Fig. 5). In this study, six OA factors, including HOA COA, CCOA, BBOA, LSOA, and RSOA were resolved after PMF analysis with ME-2. Note that OOA during SP_hs_ and NP_nhs_ is the sum of LSOA and RSOA for comparison with NP_hs_.

### Back trajectory analysis

The 3-day (72 h) back trajectories were calculated per hour at 100 m height using the Hybrid Single-Particle Lagrangian Integrated Trajectory (HYSPLIT, NOAA) 4.9 model^62,63^. The trajectories were grouped into five clusters according to Euclidean distance for weather normalization.

### Weather normalization technique

Meteorological conditions affect the variations of pollutant concentrations, which makes it difficult to directly compare pollutant emission levels. In this study, we applied a machine learning-based RF algorithm model combined with source apportionment results to decouple the effects of meteorological conditions on primary emissions and secondary formation. Detailed information on this technique can be found elsewhere^37,41^. Here, an RF model was built for each PM_1_ component and gas precursor in each year using time variables (i.e., Unix time, Julian day, month, week of the year, day of the week, hour of the day), meteorological data from observations (i.e., RH, WS, WD, temperature), meteorological data from ERA5 reanalysis data set (i.e., boundary layer height, total cloud cover, surface net solar radiation, total precipitation, and surface pressure) and air mass clusters grouped by the HYSPLIT back trajectories based on the Euclidean distance. The parameters setup for RF models was followed Vu et al.^37^. The number of trees in the random forest was 300 (n_tree = 300), the minimal node size was 3 (min_node_size = 3), and the number of variables split at each node was 3. Model performance for each pollutant during the whole study period was evaluated via Pearson’s *R*-value, root mean square error (RMSE), FAC2 (fraction of predictions with a factor of two), MB (mean bias), MGE (mean gross error), NMB (normalized mean bias), NMGE (normalized mean gross error), COE (coefficient of efficiency), and IOA (index of agreement) (as shown in Supplementary Fig. 5). For the weather normalization, only weather variables were resampled without replacement and randomly generated from the data set of different dates within a 4-week period (i.e., 2 weeks before and 2 weeks after the selected date). The selection process was repeated 1000 times to gain 1000 predicted concentrations of each species. The final weather normalized concentration of each species at a particular time was the average of that 1000 predicted results.

## Supplementary information

Supplementary Information

## Data Availability

Raw data used in this study are available from the Zenodo (10.5281/zenodo.7417822). Meteorological data, including boundary layer height, total cloud cover, surface net solar radiation, total precipitation, and surface pressure, are available from the ERA5 reanalysis data set (https://cds.climate.copernicus.eu/cdsapp#!/dataset/reanalysis-era5-single-levels?tab=overview).
