# Supplementary material for: Staggered-peak production is a mixed blessing in the control of particulate matter pollution
Source: NPJ Clim Atmos Sci. 2022 Dec 10;5(1):99. doi: 10.1038/s41612-022-00322-x (PMC9739352; doi:10.1038/s41612-022-00322-x)
Supplement: Supplementary file 1 — Supplementary Information [file 41612_2022_322_MOESM1_ESM.pdf]

Supplementary Information for

Staggered-peak production is a mixed blessing in the control of particulate matter pollution

Ying Wang[1,2], Ru-Jin Huang[1,3,4*], Wei Xu[1,6], Haobin Zhong[1,4], Jing Duan[1], Chunshui Lin[1], Yifang Gu[1,4], Ting Wang[1], Yongjie Li[5], Jurgita Ovadnevaite[6], Darius Ceburnis[6], Colin O'Dowd[6]

[1]State Key Laboratory of Loess and Quaternary Geology, Center for Excellence in Quaternary Science and Global Change, Institute of Earth Environment, Chinese Academy of Sciences, Xi'an 710061, China

[2]Interdisciplinary Research Center of Earth Science Frontier (IRCESF), Beijing Normal University, Beijing 100875, China

[3]Open Studio for Oceanic-Continental Climate and Environment Changes, Pilot National Laboratory for Marine Science and Technology (Qingdao), Qingdao 266061, China

[4]University of Chinese Academy of Sciences, Beijing 100049, China

[5]Department of Civil and Environmental Engineering, Faculty of Science and Technology, University of Macau, Taipa, Macau SAR 999078, China

[6]Ryan Institute's Centre for Climate & Air Pollution Studies, School of Natural Sciences, Physics Unit, University of Galway, University Road, Galway, H91CF50, Ireland

***Correspondence**: Ru-Jin Huang (rujin.huang@ieecas.cn)

Supplementary Information consists of 1 note, 1 table and 6 figures.

Supplementary Note 1

PMF receptor model was conducted through the interface SoFi coded in Igor Wavemetrics (Source Finder)[1]. PMF is a bilinear receptor model which enables to describe the variability of a multivariate database as the linear combination of static factor profiles and the corresponding time series:

$$\mathbf{X} = \mathbf{G} \times \mathbf{F} + \mathbf{E} \quad (1)$$

where **X** is the measurement matrix, **G** contains the factor time series, **F** is the factor profiles and **E** is the model residuals. The model uses the least squares approach to iteratively minimize the quantity **Q**, defined as the sum of the squared residuals (**eij**) weighted by their respective uncertainties (**σij**):

$$\mathbf{Q} = \sum_{i=1}^{m} \sum_{j=1}^{n} \left(\boldsymbol{e_{ij}} / \boldsymbol{\sigma_{ij}} \right)^2 \quad (2)$$

In recent years, there were lots of studies using the PMF receptor model for the OA source apportionment[2-14]. Nevertheless, the PMF receptor model usually has difficulties in separating the factors with similar profiles, such as cooking and traffic factors[15]. Therefore, the PMF receptor model was just used to help us to determine the right number of factors in this study and then the multilinear engine (ME-2) which enabled complete exploration of the rotational ambiguity by introducing *a* priori information as additional model input was used:

$$\boldsymbol{f_{j,solution}} = \boldsymbol{f_j} \pm a \times \boldsymbol{f_j} \quad (3)$$

where $\boldsymbol{f}$ refers to a row of the matrix **F**. j represents the mass to charge ratio (m/z) of the ions, and a value determines the extent to which the output profiles can differ from the model inputs and it ranges from 0 to 1.

In this work, we examined solutions from 2 to 8 factors by using the unconstrained PMF

model. According to the comparison of the mass spectra profiles and diurnal cycles as well as the analysis of time series, the 4-factor solution was the most reasonable result (Supplementary Figure 2), which can be interpreted as hydrocarbon-like OA (HOA)+cooking OA (COA), coal combustion OA (CCOA), oxygenated OA1 (OOA1) and oxygenated OA2 (OOA2). In the 4-factor PMF solution, COA and HOA were mixed as the profile had the alkyl fragment signatures which was the characteristic of HOA and the diurnal cycle showed peaks at meal time which was the characteristic of COA. And increasing the number of factors led to splitting into similar factors. Furthermore, we found significant signals for m/z 60 ($C_2H_4O_2^+$) and m/z 73 ($C_3H_5O_2^+$) which were considered as BBOA tracers in the factors HOA+COA and CCOA. And *f*60 makes up approximately 0.5% of organic mass, slightly larger than the environmental background value of 0.3%[16], indicating the existence of BBOA.

In order to separate HOA and COA in ME-2, we constrained the HOA and COA profile from Crippa et al.[17] which was derived from Paris. And we used the profile derived from Beijing winter 2014[18] to constrain BBOA. For ME-2 results with constrained HOA, COA and BBOA, one unconstrained factor was always present and was characterized by high signals at m/z 77, m/z 91, m/z 115 and m/z 44, indicating that CCOA was mixed with OOA. To separate CCOA from OOA, we constrained the CCOA profile from Wang et al.[12] which was derived from Baoji. Therefore, we finally constrained four factors (HOA, COA, CCOA, and BBOA) in the ME-2 using an *a* values between 0 and 1 with a step of 0.1 to re-adjust the input profiles and to minimize the effect of using non-local input profiles. As for all 14641 possible combinations of *a* values, a set of three criteria were established to select more environmentally meaningful results and to optimize the OA source apportionment.

1. Minimization of m/z 60 in HOA. The threshold for maximal fractional contributions of m/z 60 in HOA is 0.006 according to profiles derived from multiple ambient data sets (mean + 2σ)[19].
2. The consistency of factors with previous studies[12-14,20]. For example, profiles of OOA should have notable peaks of m/z 44 and weak signals at high m/z which is related to PAHs.
3. Optimization of COA diurnal patterns. COA has not established clear markers, so it's difficult to use COA profiles to optimize the apportionment of this source, but it's diurnal cycle can be a valuable characteristic for its identification. The COA diurnals have distinct peaks at meal times especially lunch and dinner times. Therefore, a novel approach using *k* means cluster analysis was utilized to group the normalized COA diurnals of all possible *a* value combinations. As the basis of the cost function (CF) shown in the Eq. (4), we can minimize the term $T1$ which represents the sum of the Euclidian distances between each observations (x_i) and its respective cluster center (u_{zi}) through increasing the number of clusters (k), but at the same time, the higher values of k will also lead to more complexity to the solution. Therefore, the second term ($T2$) which can be expressed as the product of the number of clusters (k) and the logarithm of the dimensionality of the cluster (D = 24 h in our case) was introduced to penalize the complexity of the higher order solutions according to Bayesian information criterion.

$$\mathrm{CF} = T1 + T2 = \|x_{\mathrm{i}} - u_{\mathrm{zi}}\|^2 + k \times \log(D). \quad (4)$$

As shown in Supplementary Figure 3, there were the cluster analysis results for four-, five-, six-cluster solutions. The left plot represented all diurnal patterns belonging to different clusters with different colors, the right plot indicated the diurnals of the cluster center. We can see from

Supplementary Figure 3 (a) that a minimum in the cost function was gotten at 5-cluster solution, which was finally chosen to be the optimal cluster number representing different types of COA diurnal patterns. From the five-cluster solution in Supplementary Figure 3 (b), the blue cluster exhibited the lowest underground concentrations and had no peaks in the nighttime. At last, 33 results meeting all three criteria were accepted. The final profiles and time series of each factor were the average from these 33 solutions which was shown in Supplementary Figure 4. Error bars in Supplementary Figure 4 are the standard deviation of each m/z.

As shown in Supplementary Figure 4, HOA was a factor closely related to motor vehicle emissions. As shown in Supplementary Figure 4, the mass spectrum of HOA in this study was highly correlated with the reference HOA mass spectra[4,6,18,19], which was characterized by alkyl fragments of $[C_nH_{2n+1}]^+$ and $[C_nH_{2n-1}]^+$, especially m/z 41, 43, 55, 57, 69, and 71[12]. There was a good correlation between the time series of HOA and its external tracer black carbon (R^2=0.61). The COA profile was similar to that of HOA, which was also characterized by hydrocarbon ion fragments such as m/z 29, 41, 43, 55, and 57. However, compared with HOA, the mass spectrum of COA had prominent ion peaks at m/z 41 and m/z 55[21] and had higher *f*41/43 (1.4) and *f*55/57 (2.7) ratio[8,17,18]. The COA profile in this study was highly correlated with that reported in Duan et al.[13] with a correlation coefficient (R^2) of 0.95, and the time series of COA was correlated well with that of m/z 55 (R^2 = 0.58). Affected by the type of coal, combustion conditions and aging process, varied mass spectra of CCOA were observed among different campaigns[12,22,23]. The CCOA mass spectrum resolved in our study was characterized by ion fragments associated with polycyclic aromatic hydrocarbons such as m/z 77, 91, and 115[9], and the time series of CCOA was correlated well with that of chloride (R^2=0.68), an external tracer

of coal combustion[24,25]. The BBOA was distinguished by the clear presence of m/z 60 ($C_2H_4O_2^+$) and m/z 73 ($C_3H_5O_2^+$) in the mass spectrum, which were known ion fragments of levoglucosan and mannan derived from pyrolysis and incomplete combustion of cellulose and hemicellulose, respectively[26]. Consistently, the time series of BBOA had a good correlation with that of m/z 60 with R^2=0.68. At the same time, the BBOA mass spectrum in this study was highly correlated with that obtained by Ng et al.[19] (R^2=0.89) based on PMF analysis results of 15 urban aerosol mass spectrometer (AMS) data sets.

Two different oxygenated OA factors were identified in 2016, namely LSOA and RSOA. The mass spectra of LSOA and RSOA both had prominent m/z 44, m/z 43 signals and weak signals at PAH-related ion peaks, while the ratio of *f*44/43 (an indicator of aging degree of factors) of RSOA (4.44) was much higher than that of LSOA (2.86), indicating that RSOA was more oxygenated than LSOA. Meanwhile, according to Supplementary Figure 5a and Supplementary Figure 5b, the time series of RSOA and LSOA also differed distinctly. RSOA had a very low mass concentration before February 26, while after February 26, the mass concentration of RSOA increased largely. In comparison, the time series of LSOA did not have this change feature, and the mass concentration of LSOA was always higher than RSOA in the entire observation. Supplementary Figure 5c showed the bivariate polar plots of LSOA and RSOA over the whole period as functions of wind directions (WD, °) and wind speeds (WS, m s^{-1}). High concentration of LSOA accumulated around the sampling site with no specific direction when the wind speed was less than 2 m s^{-1}, while the high mass concentration of RSOA appeared at high wind speed (close to 4 m s^{-1} or slightly greater than 4 m s^{-1}) which was mainly from the southeast. These characteristics indicated that the two factors were different SOA

sources and RSOA showed regional transmission features while LSOA was mainly from local formation.

Supplementary Figures

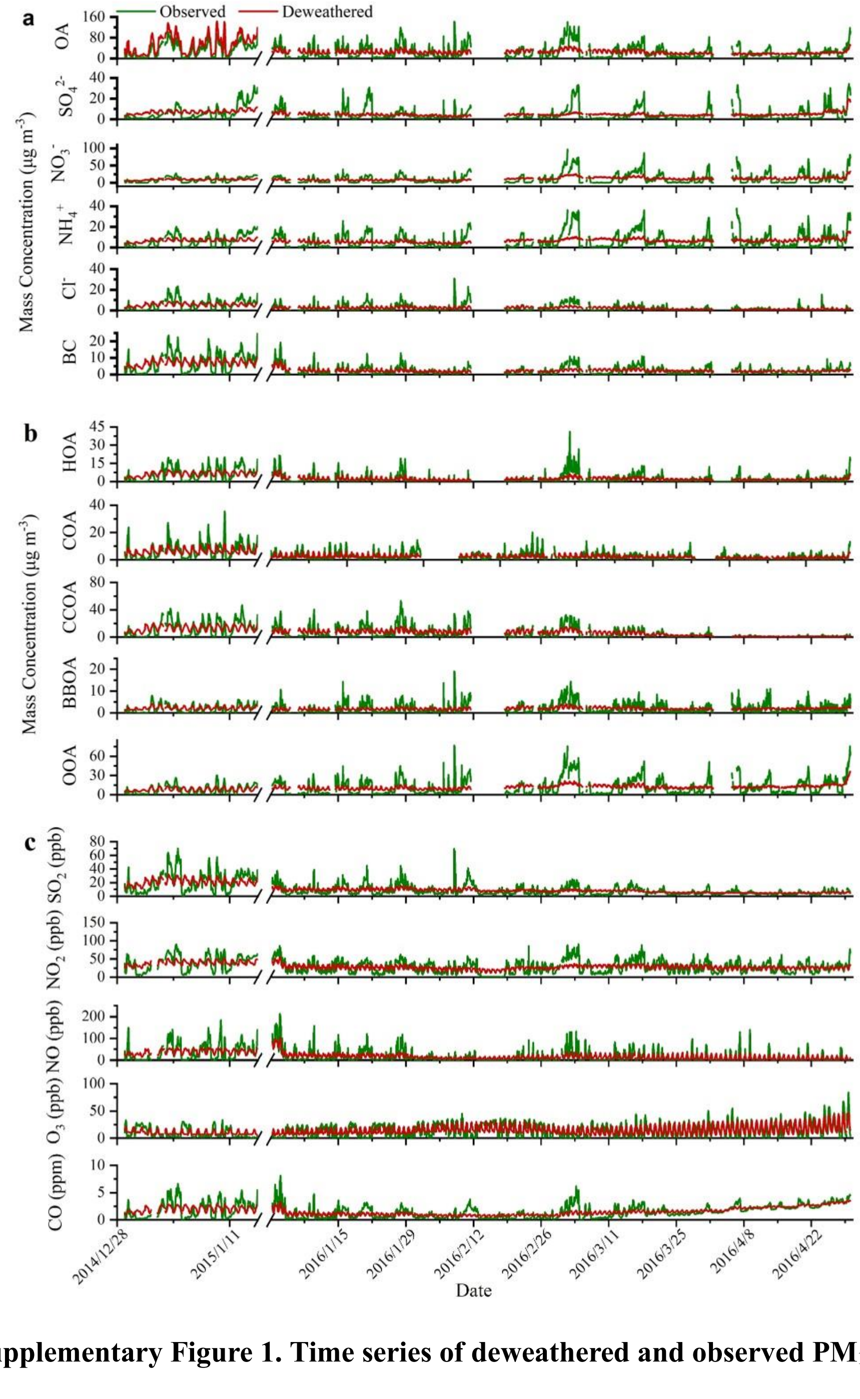

Supplementary Figure 1. Time series of deweathered and observed PM_1 species, OA factors and gaseous parameters during the whole study. Time series of **a** PM_1 species. **b** OA factors. **c** gaseous parameters.

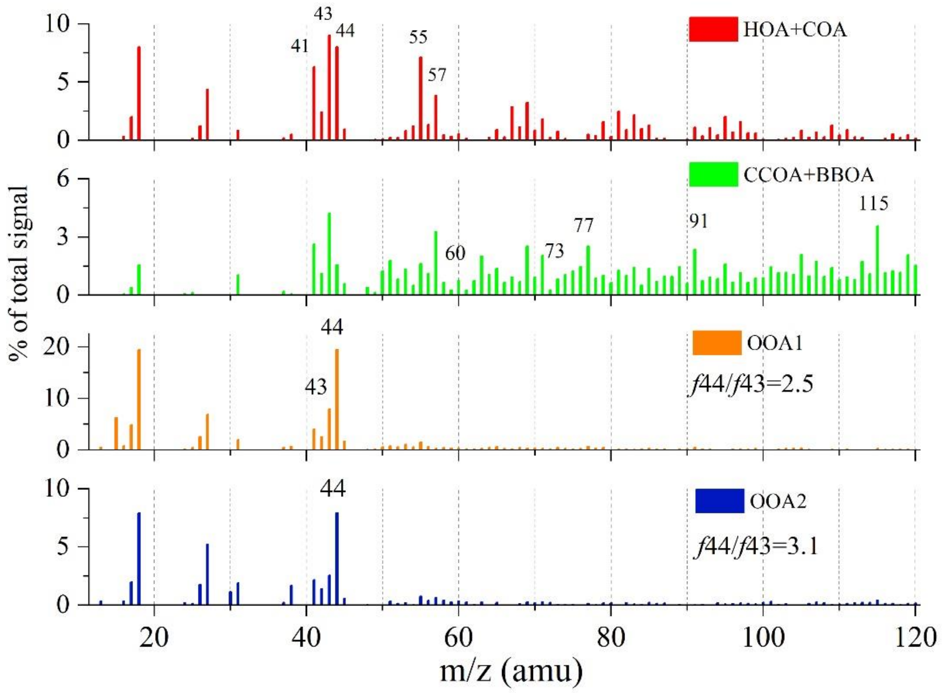

Supplementary Figure 2. Free PMF results of 4 factors.

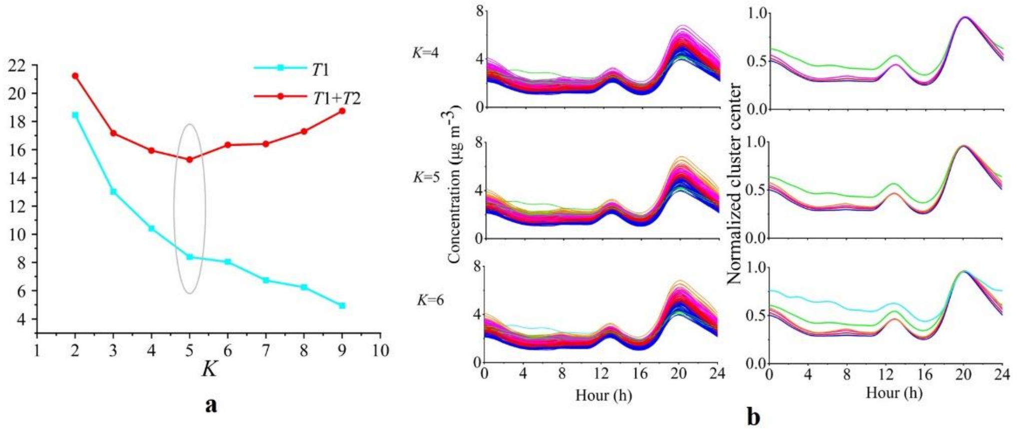

Supplementary Figure 3. Cluster analysis for the COA diurnal trends to optimize selection of a value. **a** Result of the first term (*T*1) and the total cost function defined in Eq. (4) (*T*1+ *T*2) when *k* is from 2 to 9. **b** From top to bottom are four-, five- and six-cluster results represented by different colors. For each solution, the left plots represent all COA diurnal trends colored according to responding cluster, the right plots represent the normalized COA diurnals of the

cluster center

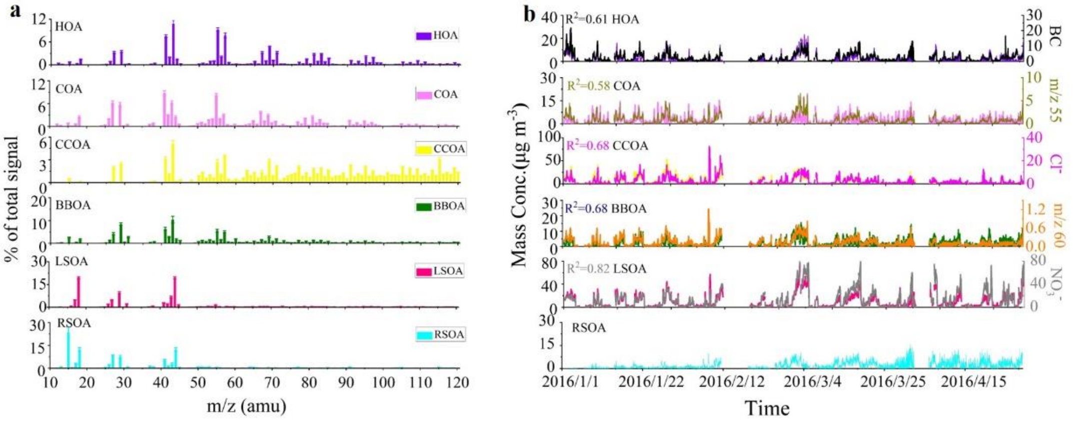

Supplementary Figure 4. Mass spectra and time series of OA factors. a Mass spectra of HOA, COA, CCOA, BBOA, LSOA and RSOA (from top to bottom). **b** Time series of HOA, COA, CCOA, BBOA, LSOA and RSOA (from top to bottom). Error bars of mass spectra represent the standard deviation of each m/z over all 33 accepted solutions. Time series of BC, m/z 55, Cl^-, m/z 60 and NO_3^- in the right plot are markers shown for supporting the source apportionment result.

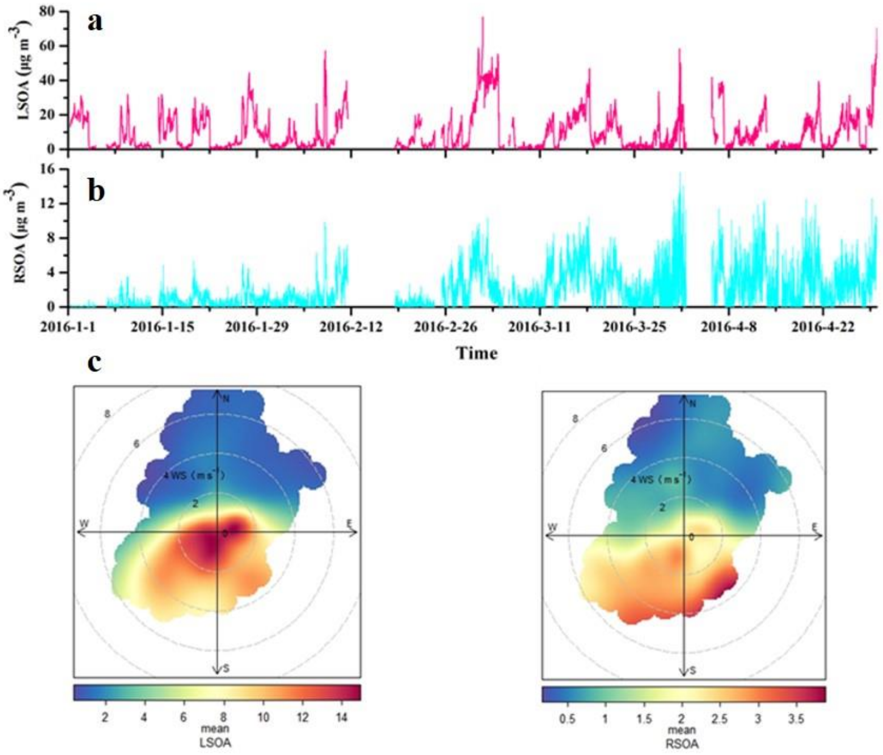

Supplementary Figure 5. Time series and bivariate polar plots of LSOA and RSOA. a Time series of LSOA for the entire study period. **b** Time series of RSOA for the entire study period. **c** Bivariate polar plots of LSOA and RSOA over the whole study period as functions of wind directions (WD) and wind speeds (WS, m s^{-1}).

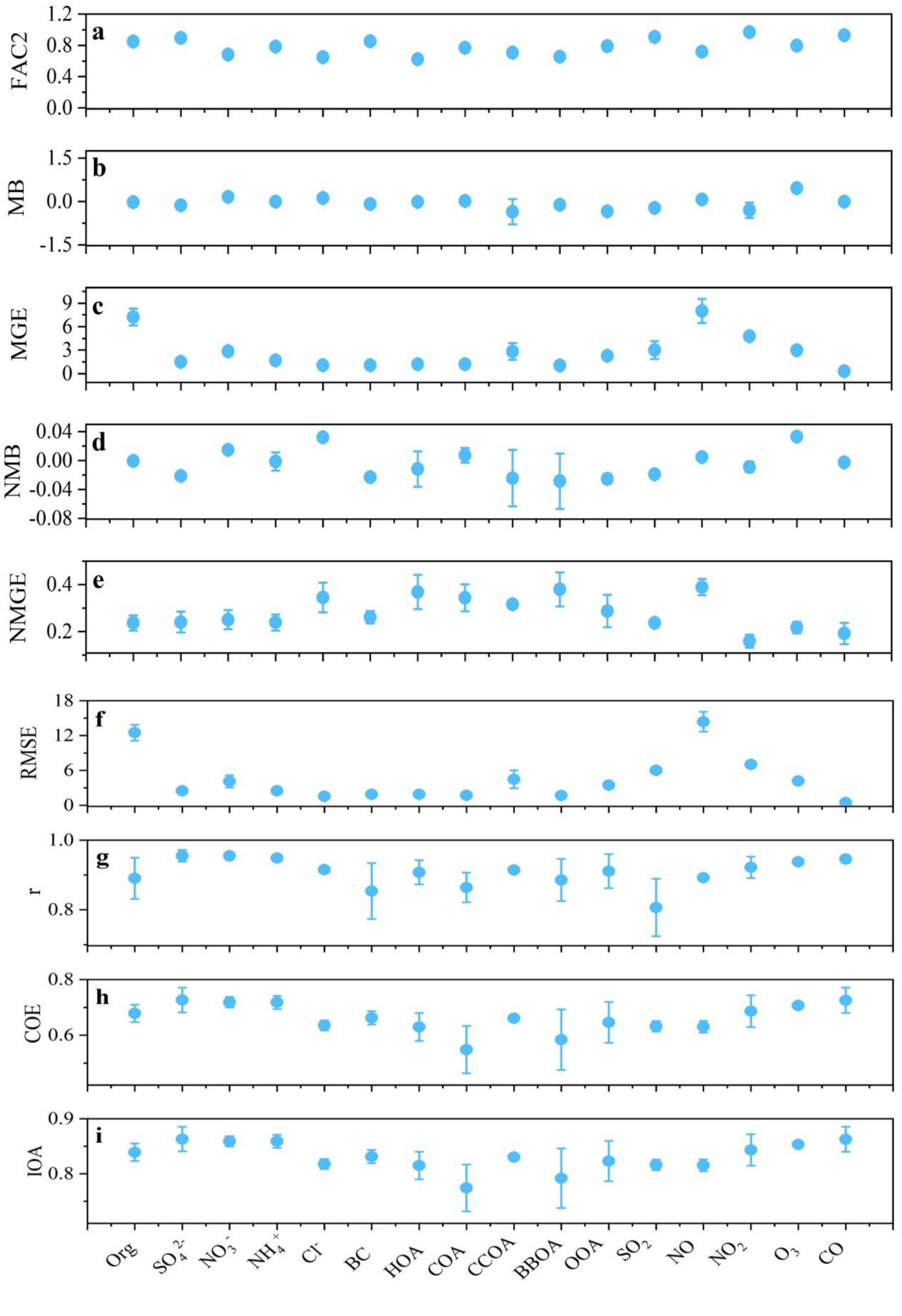

Supplementary Figure 6. The model performance parameters for testing data set of PM_1 species, OA factors, and gaseous parameters in NP_{hs}, SP_{hs} and NP_{nhs}. **a** FAC2 (fraction of predictions with a factor of two). **b** MB (mean bias). **c** MGE (mean gross error). **d** NMB. (normalized mean bias). **e** RMSE (Root-mean-square deviation). **f** NMGE (normalized mean gross error). **g** r(correlation coefficient). **h** COE (coefficient of efficiency). **i** IOA (index of

agreement). The average (± standard deviation) correlation coefficients (r) are 0.89±0.06 for organics, 0.96±0.02 for sulfate, 0.95±0.01 for nitrate, 0.95±0.01 for ammonium, 0.91±0.003 for chloride, 0.85±0.08 for BC, 0.91±0.03 for HOA, 0.86±0.04 for COA, 0.91±0.01 for CCOA, 0.89±0.06 for BBOA, 0.91±0.05 for OOA, 0.81±0.08 for SO_2, 0.89±0.01 for NO, 0.92±0.03 for NO_2, 0.94±0.01 for O_3, 0.95±0.01 for CO.

Supplementary Table

Supplementary Table 1. Observed and deweathered results under high RH conditions (RH>50%)

Species ($\mu g\ m^{-3}$)	$NP_{hs\text{-}obs}$[a]	$NP_{hs\text{-}dew}$[a]	$SP_{hs\text{-}obs}$[a]	$SP_{hs\text{-}dew}$[a]	$NP_{nhs\text{-}obs}$[a]	$NP_{nhs\text{-}dew}$[a]
SO_4^{2-}	18.4±8.0	9.1±1.6	10.7±8.1	5.3±1.2	8.1±7.5	5.5±2.6
NO_3^-	15.1±5.0	10.0±1.5	20.9±16.4	11.6±5.1	22.7±19.2	14.2±4.1
SO_2 (ppb)	29.1±6.1	21.4±2.9	15.1±8.2	10.2±1.9	6.2±2.6	5.5±1.3
NO_2 (ppb)	51.7±8.4	41.0±5.0	41.5±16.4	29.3±7.5	38.8±13.1	29.8±3.6
O_3 (ppb)	2.2±0.9	8.4±2.4	3.1±4.6	11.2±4.6	7.0±9.2	12.1±7.8
CO (ppm)	3.0±0.9	2.1±0.4	2.2±1.4	1.3±0.5	2.7±0.8	2.4±0.6
SOR	0.13±0.05	0.09±0.01	0.14±0.09	0.11±0.02	0.20±0.13	0.19±0.06
NOR	0.09±0.02	0.08±0.01	0.13±0.07	0.12±0.04	0.152±0.10	0.146±0.04

Note: [a]dew represents deweathered results; obs represents observed results.

Supplementary References

1. Canonaco, F. et al. SoFi, an Igor based interface for the efficient use of the generalized multilinear engine (ME-2) for source apportionment: Application to aerosol mass spectrometer data. *Atmos. Meas. Tech.* **6,** 6409-6443 (2013).

2. Jimenez, J. L. Ambient aerosol sampling using the Aerodyne Aerosol Mass Spectrometer. *J. Geophys. Res.* **108,** 8425 (2003).

3. Lanz, V. A. et al. Source apportionment of submicron organic aerosols at an urban site by factor analytical modelling of aerosol mass spectra. *Atmos. Chem. Phys.* **7,** 1503-1522 (2007).

4. Lanz, V. A. et al. Source attribution of submicron organic aerosols during wintertime inversions by advanced factor analysis of aerosol mass spectra. *Environ. Sci. Technol.* **42,** 214-220 (2008).

5. Jimenez, J. L. et al. Evolution of organic aerosols in the atmosphere. *Science* **326,** 1525-1529 (2009).

6. Ulbrich, I. M. et al. Interpretation of organic components from Positive Matrix Factorization of aerosol mass spectrometric data. *Atmos. Chem. Phys.* **9,** 2891-2918 (2009).

7. Ng, N. L. et al. Organic aerosol components observed in Northern Hemispheric datasets from Aerosol Mass Spectrometry. *Atmos. Chem. Phys.* **10,** 4625-4641 (2010).

8. He, L.-Y. et al. Submicron aerosol analysis and organic source apportionment in an urban atmosphere in Pearl River Delta of China using high-resolution aerosol mass spectrometry. *J. Geophys. Res.* **116,** (2011).

9. Dall'Osto, M. et al. Characterization of urban aerosol in Cork city (Ireland) using aerosol mass spectrometry. *Atmos. Chem. Phys.* **13,** 4997-5015 (2013).

10. Xu, J. et al. Chemical composition, sources, and processes of urban aerosols during summertime in northwest China: insights from high-resolution aerosol mass spectrometry. *Atmos. Chem. Phys.* **14,**

12593-12611 (2014).

11. Zhang, Y. J. et al. Insights into characteristics, sources, and evolution of submicron aerosols during harvest seasons in the Yangtze River delta region, China. *Atmos. Chem. Phys.* **15,** 1331-1349 (2015).
12. Wang, Y. C. et al. Chemical composition, sources and secondary processes of aerosols in Baoji city of northwest China. *Atmos. Environ.* **158,** 128-137 (2017).
13. Duan, J. et al. Distinctions in source regions and formation mechanisms of secondary aerosol in Beijing from summer to winter. *Atmos. Chem. Phys.* **19,** 10319-10334 (2019).
14. Huang, R.-J. et al. Primary emissions versus secondary formation of fine particulate matter in the most polluted city (Shijiazhuang) in North China. *Atmos. Chem. Phys.* **19,** 2283-2298 (2019).
15. Mohr, C. et al. Characterization of primary organic aerosol emissions from meat cooking, trash burning, and motor vehicles with high-resolution aerosol mass spectrometry and comparison with ambient and chamber observations. *Environ. Sci. Technol.* **43,** 2443-2449 (2009).
16. Cubison, M. J. et al. Effects of aging on organic aerosol from open biomass burning smoke in aircraft and laboratory studies. *Atmos. Chem. Phys.* **11,** 12049-12064 (2011).
17. Crippa, M. et al. Wintertime aerosol chemical composition and source apportionment of the organic fraction in the metropolitan area of Paris. *Atmos. Chem. Phys.* **13,** 961-981 (2013).
18. Elser, M. et al. New insights into PM2.5 chemical composition and sources in two major cities in China during extreme haze events using aerosol mass spectrometry. *Atmos. Chem. Phys.* **16,** 3207-3225 (2016).
19. Ng, N. L. et al. Real-time methods for estimating organic component mass concentrations from aerosol mass spectrometer data. *Environ. Sci. Technol.* **45,** 910-916 (2011).
20. Sun, Y. et al. Investigation of the sources and evolution processes of severe haze pollution in Beijing

in January 2013. *J. Geophys. Res. Atmos.* **119,** 4380-4398 (2014).

21. Allan, J. D. et al. Contributions from transport, solid fuel burning and cooking to primary organic aerosols in two UK cities. *Atmos. Chem. Phys.* **10,** 647-668 (2010).
22. Zhou, W., Jiang, J., Duan, L. & Hao, J. Evolution of submicrometer organic aerosols during a complete residential coal combustion process. *Environ. Sci. Technol.* **50,** 7861-7869 (2016).
23. Huang, R. et al. Contrasting sources and processes of particulate species in haze days with low and high relative humidity in wintertime Beijing. *Atmos. Chem. Phys.* **20,** 9101-9114 (2020).
24. Sun, Y. et al. Primary and secondary aerosols in Beijing in winter: sources, variations and processes. *Atmos. Chem. Phys.* **16,** 8309-8329 (2016).
25. Li, H. et al. Wintertime aerosol chemistry and haze evolution in an extremely polluted city of the North China Plain: significant contribution from coal and biomass combustion. *Atmos. Chem. Phys.* **17,** 4751-4768 (2017).
26. Alfarra, M. R. et al. Identification of the mass spectral signature of organic aerosols from wood burning emissions. *Environ. Sci. Technol.* **41,** 5770-5777 (2007).
